# The therapeutic potential of genome editing for β-thalassemia

**DOI:** 10.12688/f1000research.7087.1

**Published:** 2015-12-11

**Authors:** Astrid Glaser, Bradley McColl, Jim Vadolas

**Affiliations:** 1 1Murdoch Childrens Research Institute, Royal Children’s Hospital, 50 Flemington Rd, Parkville, VIC, 3052, Australia; 2 Department of Paediatrics, University of Melbourne, Royal Children's Hospital, 50 Flemington Rd, Parkville, VIC, 3052, Australia

**Keywords:** thalassemia, genome, gene therapy

## Abstract

The rapid advances in the field of genome editing using targeted endonucleases have called considerable attention to the potential of this technology for human gene therapy. Targeted correction of disease-causing mutations could ensure lifelong, tissue-specific expression of the relevant gene, thereby alleviating or resolving a specific disease phenotype. In this review, we aim to explore the potential of this technology for the therapy of β-thalassemia. This blood disorder is caused by mutations in the gene encoding the β-globin chain of hemoglobin, leading to severe anemia in affected patients. Curative allogeneic bone marrow transplantation is available only to a small subset of patients, leaving the majority of patients dependent on regular blood transfusions and iron chelation therapy. The transfer of gene-corrected autologous hematopoietic stem cells could provide a therapeutic alternative, as recent results from gene therapy trials using a lentiviral gene addition approach have demonstrated. Genome editing has the potential to further advance this approach as it eliminates the need for semi-randomly integrating viral vectors and their associated risk of insertional mutagenesis. In the following pages we will highlight the advantages and risks of genome editing compared to standard therapy for β-thalassemia and elaborate on lessons learned from recent gene therapy trials.

## β-Thalassemia

β-Thalassemia is a common congenital blood disorder caused by mutations in the β-globin gene. Reduced or absent β-globin expression leads to an imbalance of the α-globin and β-globin subunits that form the hemoglobin tetramer. The toxic accumulation of excess α-globin chains in developing erythrocytes results in severe anemia due to ineffective erythropoiesis ^1^. In its most serious form, β-thalassemia major, the condition is fatal if left untreated ^2^. Currently, allogenic bone marrow transplantation (BMT) is the only curative therapeutic option. However, due to the rarity of suitable donors, this treatment is available only to a small subset of patients and the procedure itself entails a risk of potentially life-threatening immunological complications and graft failure, especially for patients over 3 years of age ^3, 4^.

The majority of β-thalassemia patients depend on regular blood transfusions combined with iron chelation therapy for their survival ^5^. Even under optimal care, this treatment regimen provides a suboptimal quality of life and leaves patients at an increased risk of death from cardiomyopathies and infection ^6, 7^. New therapeutic strategies are therefore needed to better manage β-thalassemia.

## Gene therapy for β-Thalassemia

In the past 25 years, the field of gene therapy has made considerable progress. Gene therapy aims at the functional cure of disorders through modification of a patient’s genome. Depending on the nature of the causative mutation, this could be achieved through the introduction of a therapeutic gene, correction of the disease-causing mutation, or the elimination of deleterious gene products (reviewed by Kay *et al.*, 2011) ^8^.

The major obstacle all gene therapy approaches face is safe and efficient gene delivery to the affected tissue or cell type. *In vivo* delivery is particularly difficult due to poor tissue accessibility, vector immunogenicity, and limited target cell specificity ^9^. Monogenic blood disorders such as severe combined immune deficiency (SCID), sickle-cell anemia, and β-thalassemia are remarkably attractive targets for gene therapy due to the unique accessibility of hematopoietic progenitor cells, which can be isolated from patient bone marrow ^10^. The *ex vivo* correction and re-introduction of autologous hematopoietic stem cells (HSCs) has no associated risk of graft-versus-host disease, the major adverse effect of allogenic BMT. Eliminating the necessity of a matched donor potentially makes this approach applicable to all patients. Gene therapy could therefore provide a safer and more generally available curative treatment for blood disorders than allogenic BMT.

Past and ongoing gene therapy trials are mostly focused on the delivery of a therapeutic gene using integrating viral vectors. This gene addition approach has been successfully applied in severe combined immunodeficiencies ^11– 13^, retinal disorders ^14– 16^, and hemophilia ^17^. The first successful gene therapy trial for β-thalassemia was reported in 2010 ^18^. The trial employed a lentiviral vector for *ex vivo* delivery of a β-globin transgene into patient HSCs, which were subsequently returned to the patient. The treatment was successful in one patient who remained transfusion-independent for up to 7 years ^19, 20^. A second trial was subsequently initiated using a modified vector. Although long-term results are yet to be released, promising preliminary data describe two patients remaining transfusion-independent for 14 and 16 months, respectively ^21^. These trials demonstrate that gene therapy has the potential to provide effective long-term therapy following a single treatment.

The greatest caveat in the use of integrating lentiviral and retroviral vectors lies in the inability to control for target site selection, which can result in considerable genotoxicity from the transactivation of nearby proto-oncogenes ^22, 23^. This was tragically confirmed when four out of nine children treated in the first gene therapy trial for SCID-X1 developed leukemia as a result of gamma-retrovirus vector integration, causing the death of one patient ^24^. Following this setback, vector design was improved by the development of self-inactivating lentiviruses, insulator elements, and tissue-specific promoters ^25– 27^. Nonetheless, insertional mutagenesis still remains the major concern with retroviral and lentiviral gene therapy approaches ^28^. The importance of understanding and managing this risk was again demonstrated by the appearance of a dominant clone with a transactivating insertion event near the HMGA2 gene in the HSCs of the first successfully treated β-thalassemia gene therapy patient ^18^. This event, though only transient, has again emphasized the necessity for careful monitoring of patients following treatment with integrating vectors.

This issue has driven the search for safer gene therapy approaches. One possible solution is the targeted integration of a therapeutic gene into a genomic “safe harbor” site that supports long-term transgene expression without affecting transcriptional activity at endogenous loci. The natural preference of adeno-associated viruses (AAVs) for integration at the AAVS1 site on chromosome 19 could potentially provide an alternative to the semi-random integration profile of lentiviral and retroviral vectors ^29^. However, their small transgene capacity limits the usefulness of AAVs as gene therapy vectors ^30– 32^. Hybrid strategies combining the site-selective recombinase activity of the AAV rep protein with larger vectors have the potential to overcome this limitation. Based on this principle, we have previously achieved targeted integration of a bacterial artificial chromosome carrying the whole human β-globin locus into the AAVS1 site in K562 cells ^33^. Another approach, gene repair through homologous recombination, has been proposed already in the 1980s ^34, 35^. In 1985, Smithies *et al.* demonstrated the introduction of heterologous DNA sequences into the β-globin locus of human cell lines using homologous recombination ^36^. These results led to the first speculation that targeted genome modification via homologous recombination in HSCs could provide a cure for β-hemoglobinopathies. However, before the emergence of targeted endonucleases, this approach remained limited by low efficiency.

## Genome editing

**Figure 1.**
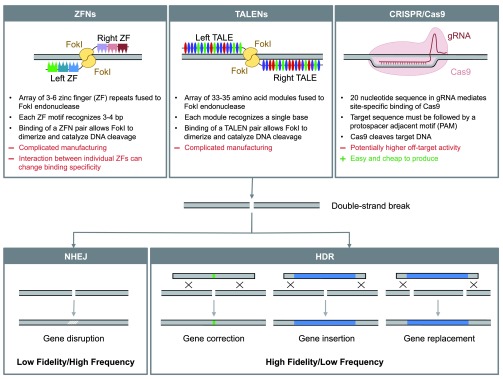
Genome editing technologies. ZFNs, TALENs, and CRISPR/Cas9 are used to introduce site-specific DSBs into a target genome. Subsequently, cellular repair mechanisms can be harnessed to introduce precise genetic modifications. Small insertions and deletions generated by NHEJ can be used for gene knockout. In the presence of a homologous repair template, new sequences can be incorporated via HDR, allowing for gene repair, transgene insertion, and gene replacement.

The discovery and development of targetable endonucleases has kindled a new enthusiasm for the previously niche area of genome modification through homologous repair. These enzymes can be engineered to introduce a site-specific double-strand break (DSB) into a target genome, which can subsequently be repaired by endogenous DNA repair mechanisms ( Figure 1). Mammalian cells possess two major DSB repair pathways: non-homologous end-joining (NHEJ) and homology-directed repair (HDR) ^37, 38^. NHEJ is error prone and leads to the creation of small insertions or deletions at the DSB site. This has been used very efficiently for targeted gene knockout in a variety of cell types and for the generation of knockout animal models ^39– 44^. HDR uses a homologous DNA template to repair the broken strand with high fidelity. Fusion of a reporter to a gene of interest and gene insertion, as well as targeted gene correction, have been demonstrated using this approach ^45– 47^.

**Figure 2.**
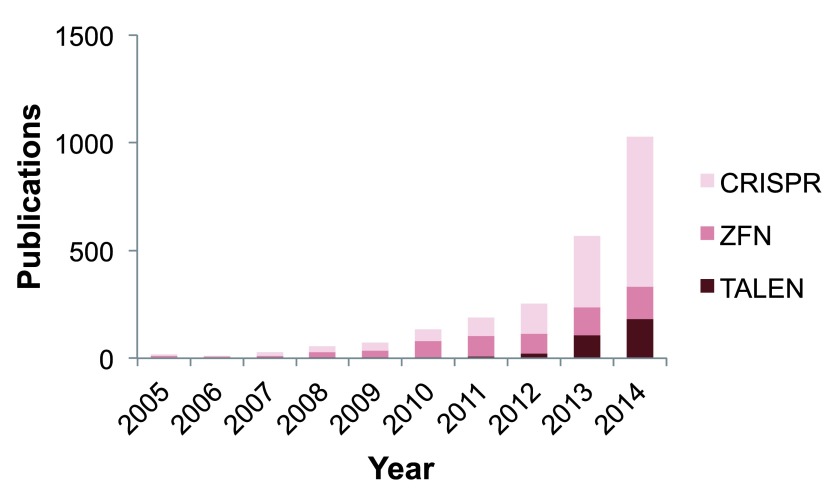
Publications on genome editing between 2005 and 2014. Data obtained from Medline trend using the search terms “CRISPR Cas9”, “Zinc-finger nuclease”, and “TALEN” show an increase in the use of programmable endonucleases during this period ^99^.

There are three different types of programmable endonucleases. Zinc-finger nucleases (ZFNs) and transcription activator-like effector nucleases (TALENs) are generated by the fusion of repetitive arrays of specific DNA-binding amino acid motifs to a FokI endonuclease domain ( Figure 1) ^48– 50^. Binding of a pair of ZFNs or TALENs on opposite DNA strands allows their FokI domains to dimerize and become catalytically active, introducing a DSB with 5’ overhangs into the target site. ZFNs and TALENs both require complicated cloning approaches to achieve the arrangement of repetitive motifs, imposing penalties of time and expense upon their development. The recent adaptation of clustered-interspaced short palindromic repeats (CRISPRs) and CRISPR-associated protein 9 (Cas9) for use in mammalian cells has greatly facilitated genome editing applications. The Cas9 endonuclease is targeted to a specific DNA sequence by a complementary 20-nucleotide sequence in a guide RNA (gRNA) bound to the Cas9 protein. New gRNAs can be generated quickly and at low cost using standard cloning techniques ^51, 52^. Due to the ease of use of the Cas9 system, its use has rapidly surpassed that of ZFNs and TALENs in the past years ( Figure 2).

Similar to lentiviral gene therapy, genome editing could be used to correct patient HSCs *ex vivo* for the gene therapy of β-thalassemia. Ideally, scarless correction of the β-globin gene in HSCs could be achieved through HDR, resulting in the production of healthy erythrocytes. Several studies have shown that the human β-globin locus is amenable to genome editing ( Table 1) ^53– 61^. However, technical limitations and safety concerns need to be overcome for this novel approach to become clinically applicable.

**Table 1.** Recent studies employing novel strategies for therapeutic genome editing at the human β-globin locus. (iPSCs: induced pluripotent stem cells).

Strategy	Cell type	Platform	Reference
Correction of β-thalassemia mutations	Patient iPSCs	CRISPR/Cas9	Xie *et al.*, 2014 ^55^
	Patient iPSCs	TALENS	Ma *et al.*, 2013 ^57^
Correction of sickle-cell mutation	Patient iPSCs	TALENs	Sun *et al.*, 2014 ^62^
	Patient iPSCs	TALENs	Ramalingam *et al.*, 2014 ^58^
	Patient iPSCs	CRISPR/Cas9	Huang *et al.*, 2015 ^54^
	HSCs	ZFNs	Hoban *et al.*, 2015 ^60^
Gene insertion of β-globin cDNA	K562	TALENs	Voit *et al.*, 2014 ^53^
γ-globin reactivation	MEL	TALENs	Wienert *et al.*, 2015 ^59^

In contrast to viral gene addition approaches, genome editing does not require the use of integrating vectors, as transient expression of a targeted endonuclease is sufficient to achieve the necessary DNA cleavage. This eliminates the issue of insertional mutagenesis. However, off-target cleavage at sites other than that intended is a major concern with genome editing approaches ^63– 65^. For the CRISPR/Cas9 system, strategies have been developed to reduce the relatively high off-target cleavage associated with wild-type Cas9. A mutated Cas9 protein that introduces a single-stranded nick rather than a DSB can be used to increase cleavage specificity. Consequently, two gRNAs designed to mediate nicking on opposite strands at the target site are required to form a DSB ^63^. However, a single gRNA is still sufficient to introduce a DNA nick at off-target sites, which may have adverse effects in the target cell. Alternatively, an inactive Cas9 protein can be fused to FokI, which only becomes enzymatically active upon dimerization. With this approach, two Cas9/FokI hybrid units need to be brought together by specific gRNAs to allow cleavage at the target site ^66, 67^. The target specificity of Cas9 can be further increased through the use of a truncated guide sequence of 17 instead of 20 nucleotides ^68^. However, off-target activity of any nuclease type still varies between different genomic targets and cell types ^69, 70^. Therefore, as with all gene therapy strategies, careful vector design and thorough evaluation of risks is necessary.

Off-target site prediction tools that rank potential unintended cleavage sites based on similarity scores were developed to facilitate the evaluation of cleavage stringency for different nuclease platforms. It remains to be determined if the targeted analysis of selected putative off-target sites is sufficient for the determination of nuclease-associated risks. Further validation of the reliability of these prediction tools via unbiased genome-wide detection of off-target cleavage is therefore required. Approaches taking advantage of the occasional capture of foreign sequences in genomic DSBs show promise to close this information gap ^71– 73^. Many studies report minimal to no detectable off-target activity across a variety of nuclease platforms and target sites ^60, 66, 67, 74– 77^. A 2015 publication using ZFNs to correct the sickle-cell mutation in primary patient HSCs indicates that therapeutic genome editing of the β-globin gene can be achieved without producing deleterious unintended mutations. The only off-target events detected in a genome-wide analysis were located in the highly homologous δ-globin gene, which is non-essential ^60^. Also, the first clinical phase I human genome editing trial using ZFNs to disrupt the CCR5 co-receptor for HIV entry in autologous CD4 T cells has not produced any adverse events that could be attributed to the use of ZFNs ^78^. While further confirmation is still required, these findings suggest that off-target effects will not restrict genome editing from clinical applications.

Gene therapy trials for SCID are simplified due to the selective advantage of gene corrected cells over unmodified HSCs ^11^. In the case of the β-hemoglobinopathies, β-globin expression does not convey an advantage for HSCs. Consequently, a substantial fraction of HSCs needs to be modified to achieve a therapeutic effect. Lentiviral or retroviral delivery and nucleofection of DNA or mRNA can achieve transfection rates greater than 80% in primary human HSCs ^79– 81^. These methods are also suitable for the delivery of genome editing tools. A high transduction efficiency, leading to a high frequency of target cleavage, is essential for efficient genome editing. However, low HDR frequency in naïve HSCs, accompanied by a background of disruptive NHEJ, currently impedes the generation of therapeutic levels of edited cells ^60, 82^. Although NHEJ is unlikely to produce adverse effects in an already non-functional gene, it will be crucial to increase the fraction of cells that undergo HDR genome editing to be successful in the clinic. Several groups have developed screening methods that permit simultaneous quantification of NHEJ and HDR ^83– 87^. These can be used for the identification of conditions that favor HDR. Most notably, inhibition of DNA ligase 4, which is required for the NHEJ pathway, has been shown to not only decrease NHEJ but also increase HDR frequencies in cell lines and mouse embryos ^88, 89^. As the repair pathway choice in a cell is largely dependent on the cell cycle stage, cell synchronization and timed nuclease delivery could also bias cells towards HDR ^90^. Increasing the frequency of gene correction in HSCs will be crucial in determining the feasibility of therapeutic genome editing in the hematopoietic system.

## A future in the clinic

Although low HDR efficiency and safety concerns regarding off-target effects are currently obstructing the therapeutic application of genome editing, strategies to resolve these limitations are rapidly progressing. As with all novel therapeutics, every custom genome editing vector will be subject to careful clinical trials. It is therefore crucial to design therapeutic genome editing strategies to be as inclusive as possible, i.e. to minimize the number of different vectors required to treat the maximum number of patients. While over 200 mutations are known to cause β-thalassemia, a relatively small number of mutations account for the majority of cases ^91^. Therefore, a small number of Cas9/gRNA vectors could be sufficient to address the majority of patients. Alternatively, the introduction of two DSBs at either side of the β-globin gene could allow for gene replacement without the need for allele-specific vectors, thus placing a therapeutic β-globin under the control of endogenous regulatory elements at the β-globin locus ^53, 92^. Like lentiviral gene therapy, genome editing can also be applied to gene addition. A single genome editing vector targeting a safe harbor site could be combined with a separate HDR template containing a therapeutic β-globin gene. This approach has the potential to provide a universally applicable strategy, as a single genome editing vector could be used for a large range of monogenic disorders by simply exchanging the HDR template. Genome editing also has the potential to introduce mutations that modify the severity of β-thalassemia. It is known from individuals with hereditary persistence of fetal hemoglobin that elevated expression of γ-globin, a developmentally silenced β-globin-like gene, can be protective of the pathologic effects associated with the absence of β-globin expression ^93, 94^. Replication of this phenotype through genome editing could therefore alleviate the symptoms in β-thalassemic patients. A recent study employed TALENs to introduce a single point mutation within the β-globin locus to increase the expression of γ-globin ^59^. Interference with the expression of BCL11A, a major regulator of β-globin gene expression, has also been shown to promote the expression of γ-globin ^95, 96^. An erythroid-specific enhancer for BCL11A expression was recently identified by Bauer *et al.* ^97^. Targeted elimination of this enhancer in patient-derived HSCs could allow the induction of γ-globin expression in erythroid cells without affecting BCL11A-dependent processes in other lineages ^98^. This could be achieved through an NHEJ approach, unimpeded by the low frequency that currently limits strategies depending on HDR. However, in the future, the latter could be applied to the correction of patient-derived iPSCs, thus circumventing the issue of HDR efficiency, since a large number of cells can be generated from a few corrected clones. With this range of possibilities, genome editing is diversifying gene therapy research with the potential to greatly relieve the global health burden of the β-hemoglobinopathies.

## Abbreviations

AAV, adeno-associated virus; BMT, bone marrow transplantation; Cas9, CRISPR-associated protein 9; CRISPRs, clustered interspaced palindromic repeats; DSB, double strand break; gRNA, guide RNA; HDR, homology-directed repair; HSCs, hematopoietic stem cells; iPSCs induced pluripotent stem cells; NHEJ, non-homologous end-joining; TALENs, transcription activator-like effector nucleases; SCID, severe combined immune deficiency; ZFNs, zinc-finger nucleases

## References

[1] Ribeil JA Arlet JB Dussiot M: Ineffective erythropoiesis in β -thalassemia. *ScientificWorldJournal.* 2013;2013:394295. 10.1155/2013/394295 23606813 PMC3628659

[2] Lanzkowsky P: Manual of pediatric hematology and oncology.2011;5 Reference Source

[3] King A Shenoy S: Evidence-based focused review of the status of hematopoietic stem cell transplantation as treatment of sickle cell disease and thalassemia. *Blood.* 2014;123(20):3089–94; quiz 3210. 10.1182/blood-2013-01-435776 24511087

[4] Gennery AR Slatter MA Grandin L: Transplantation of hematopoietic stem cells and long-term survival for primary immunodeficiencies in Europe: entering a new century, do we do better? *J Allergy Clin Immunol.* 2010;126(3):602–10.e1-11. 10.1016/j.jaci.2010.06.015 20673987

[5] Galanello R Origa R: Beta-thalassemia. *Orphanet J Rare Dis.* 2010;5:11. 10.1186/1750-1172-5-11 20492708 PMC2893117

[6] Kremastinos DT Farmakis D Aessopos A: Beta-thalassemia cardiomyopathy: history, present considerations, and future perspectives. *Circ Heart Fail.* 2010;3(3):451–8. 10.1161/CIRCHEARTFAILURE.109.913863 20484195

[7] Cappellini MD Cohen A Eleftheriou A: Guidelines for the Clinical Management of Thalassaemia [Internet].2008. 24308075

[8] Kay MA: State-of-the-art gene-based therapies: the road ahead. *Nat Rev Genet.* 2011;12(5):316–28. 10.1038/nrg2971 21468099

[9] Mali S: Delivery systems for gene therapy. *Indian J Hum Genet.* 2013;19(1):3–8. 10.4103/0971-6866.112870 23901186 PMC3722627

[10] Herzog RW Hagstrom JN: Gene therapy for hereditary hematological disorders. *Am J Pharmacogenomics.* 2001;1(2):137–44. 12174674 10.2165/00129785-200101020-00006

[11] Fischer A Hacein-Bey Abina S Touzot F: Gene therapy for primary immunodeficiencies. *Clin Genet.* 2015;88(6):507–15. 10.1111/cge.12576 25708106

[12] Hacein-Bey-Abina S Hauer J Lim A: Efficacy of gene therapy for X-linked severe combined immunodeficiency. *N Engl J Med.* 2010;363(4):355–64. 10.1056/NEJMoa1000164 20660403 PMC2957288

[13] Gaspar HB Cooray S Gilmour KC: Hematopoietic stem cell gene therapy for adenosine deaminase-deficient severe combined immunodeficiency leads to long-term immunological recovery and metabolic correction. *Sci Transl Med.* 2011;3(97):97ra80. 21865538 10.1126/scitranslmed.3002716

[14] Bainbridge JW Smith AJ Barker SS: Effect of gene therapy on visual function in Leber's congenital amaurosis. *N Engl J Med.* 2008;358(21):2231–9. 10.1056/NEJMoa0802268 18441371

[15] Hauswirth WW Aleman TS Kaushal S: Treatment of leber congenital amaurosis due to RPE65 mutations by ocular subretinal injection of adeno-associated virus gene vector: short-term results of a phase I trial. *Hum Gene Ther.* 2008;19(10):979–90. 10.1089/hum.2008.107 18774912 PMC2940541

[16] Maguire AM Simonelli F Pierce EA: Safety and efficacy of gene transfer for Leber's congenital amaurosis. *N Engl J Med.* 2008;358(21):2240–8. 10.1056/NEJMoa0802315 18441370 PMC2829748

[17] Nathwani AC Tuddenham EG Rangarajan S: Adenovirus-associated virus vector-mediated gene transfer in hemophilia B. *N Engl J Med.* 2011;365(25):2357–65. 10.1056/NEJMoa1108046 22149959 PMC3265081

[18] Cavazzana-Calvo M Payen E Negre O: Transfusion independence and *HMGA2* activation after gene therapy of human β-thalassaemia. *Nature.* 2010;467(7313):318–22. 10.1038/nature09328 20844535 PMC3355472

[19] Press release. First two patients in the HGB-205 Study achieved transfusion independence within two weeks of an autologous transplant with bluebird’s lentiviral gene therapy.2014 Reference Source

[20] Finotti A Breda L Lederer CW: Recent trends in the gene therapy of β-thalassemia. *J Blood Med.* 2015;6:69–85. 10.2147/JBM.S46256 25737641 PMC4342371

[21] Press Release. Bluebird Bio Reports New Beta-thalassemia Major and Severe Sickle Cell Disease Data from HGB-205 Study at EHA.2015 Reference Source

[22] Trobridge GD: Genotoxicity of retroviral hematopoietic stem cell gene therapy. *Expert Opin Biol Ther.* 2011;11(5):581–93. 10.1517/14712598.2011.562496 21375467 PMC3443588

[23] Hacein-Bey-Abina S Von Kalle C Schmidt M: *LMO2*-associated clonal T cell proliferation in two patients after gene therapy for SCID-X1. *Science.* 2003;302(5644):415–9. 10.1126/science.1088547 14564000

[24] Hacein-Bey-Abina S Garrigue A Wang GP: Insertional oncogenesis in 4 patients after retrovirus-mediated gene therapy of SCID-X1. *J Clin Invest.* 2008;118(9):3132–42. 10.1172/JCI35700 18688285 PMC2496963

[25] Emery DW: The use of chromatin insulators to improve the expression and safety of integrating gene transfer vectors. *Hum Gene Ther.* 2011;22(6):761–74. 10.1089/hum.2010.233 21247248 PMC3107579

[26] Yi Y Hahm SH Lee KH: Retroviral gene therapy: safety issues and possible solutions. *Curr Gene Ther.* 2005;5(1):25–35. 10.2174/1566523052997514 15638709

[27] Zhou S Mody D DeRavin SS: A self-inactivating lentiviral vector for SCID-X1 gene therapy that does not activate LMO2 expression in human T cells. *Blood.* 2010;116(6):900–8. 10.1182/blood-2009-10-250209 20457870 PMC2924228

[28] Wu C Dunbar CE: Stem cell gene therapy: the risks of insertional mutagenesis and approaches to minimize genotoxicity. *Front Med.* 2011;5(4):356–71. 10.1007/s11684-011-0159-1 22198747 PMC3508510

[29] Samulski RJ Zhu X Xiao X: Targeted integration of adeno-associated virus (AAV) into human chromosome 19. *EMBO J.* 1991;10(12):3941–50. 1657596 10.1002/j.1460-2075.1991.tb04964.x PMC453134

[30] Dong JY Fan PD Frizzell RA: Quantitative analysis of the packaging capacity of recombinant adeno-associated virus. *Hum Gene Ther.* 1996;7(17):2101–12. 10.1089/hum.1996.7.17-2101 8934224

[31] Grieger JC Samulski RJ: Packaging capacity of adeno-associated virus serotypes: impact of larger genomes on infectivity and postentry steps. *J Virol.* 2005;79(15):9933–44. 10.1128/JVI.79.15.9933-9944.2005 16014954 PMC1181570

[32] Hermonat PL Quirk JG Bishop BM: The packaging capacity of adeno-associated virus (AAV) and the potential for *wild-type-plus* AAV gene therapy vectors. *FEBS Lett.* 1997;407(1):78–84. 10.1016/S0014-5793(97)00311-6 9141485

[33] Howden SE Voullaire L Wardan H: Site-specific, Rep-mediated integration of the intact beta-globin locus in the human erythroleukaemic cell line K562. *Gene Ther.* 2008;15(20):1372–83. 10.1038/gt.2008.84 18496574

[34] Vega MA: Prospects for homologous recombination in human gene therapy. *Hum Genet.* 1991;87(3):245–53. 10.1007/BF00200899 1864597

[35] Capecchi MR: Altering the genome by homologous recombination. *Science.* 1989;244(4910):1288–92. 10.1126/science.2660260 2660260

[36] Smithies O Gregg RG Boggs SS: Insertion of DNA sequences into the human chromosomal beta-globin locus by homologous recombination. *Nature.* 1985;317(6034):230–4. 10.1038/317230a0 2995814

[37] Chapman JR Taylor MR Boulton SJ: Playing the end game: DNA double-strand break repair pathway choice. *Mol Cell.* 2012;47(4):497–510. 10.1016/j.molcel.2012.07.029 22920291

[38] Zhang Y Vanoli F LaRocque JR: Biallelic targeting of expressed genes in mouse embryonic stem cells using the Cas9 system. *Methods.* 2014;69(2):171–8. 10.1016/j.ymeth.2014.05.003 24929070 PMC4405113

[39] Bauer DE Canver MC Orkin SH: Generation of genomic deletions in mammalian cell lines via CRISPR/Cas9. *J Vis Exp.* 2015; (95). 10.3791/52118 25549070 PMC4279820

[40] Brandl C Ortiz O Röttig B: Creation of targeted genomic deletions using TALEN or CRISPR/Cas nuclease pairs in one-cell mouse embryos. *FEBS Open Bio.* 2015;5:26–35. 10.1016/j.fob.2014.11.009 25685662 PMC4309836

[41] Peng D Kurup SP Yao PY: CRISPR-Cas9-mediated single-gene and gene family disruption in *Trypanosoma cruzi*. *MBio.* 2015;6(1):e02097–14. 10.1128/mBio.02097-14 25550322 PMC4281920

[42] Gratz SJ Wildonger J Harrison MM: CRISPR/Cas9-mediated genome engineering and the promise of designer flies on demand. *Fly (Austin).* 2013;7(4):249–55. 10.4161/fly.26566 24088745 PMC3896497

[43] Mandal PK Ferreira LM Collins R: Efficient ablation of genes in human hematopoietic stem and effector cells using CRISPR/Cas9. *Cell Stem Cell.* 2014;15(5):643–52. 10.1016/j.stem.2014.10.004 25517468 PMC4269831

[44] Wang W Ye C Liu J: *CCR5* gene disruption via lentiviral vectors expressing Cas9 and single guided RNA renders cells resistant to HIV-1 infection. *PLoS One.* 2014;9(12):e115987. 10.1371/journal.pone.0115987 25541967 PMC4277423

[45] Wang H Yang H Shivalila CS: One-step generation of mice carrying mutations in multiple genes by CRISPR/Cas-mediated genome engineering. *Cell.* 2013;153(4):910–8. 10.1016/j.cell.2013.04.025 23643243 PMC3969854

[46] Yin H Xue W Chen S: Genome editing with Cas9 in adult mice corrects a disease mutation and phenotype. *Nat Biotechnol.* 2014;32(6):551–3. 10.1038/nbt.2884 24681508 PMC4157757

[47] Li HL Nakano T Hotta A: Genetic correction using engineered nucleases for gene therapy applications. *Dev Growth Differ.* 2014;56(1):63–77. 10.1111/dgd.12107 24329887

[48] Kim YG Cha J Chandrasegaran S: Hybrid restriction enzymes: zinc finger fusions to Fok I cleavage domain. *Proc Natl Acad Sci U S A.* 1996;93(3):1156–60. 8577732 10.1073/pnas.93.3.1156 PMC40048

[49] Boch J Scholze H Schornack S: Breaking the code of DNA binding specificity of TAL-type III effectors. *Science.* 2009;326(5959):1509–12. 10.1126/science.1178811 19933107

[50] Christian M Cermak T Doyle EL: Targeting DNA double-strand breaks with TAL effector nucleases. *Genetics.* 2010;186(2):757–61. 10.1534/genetics.110.120717 20660643 PMC2942870

[51] Mali P Yang L Esvelt KM: RNA-guided human genome engineering via Cas9. *Science.* 2013;339(6121):823–6. 10.1126/science.1232033 23287722 PMC3712628

[52] Cong L Ran FA Cox D: Multiplex genome engineering using CRISPR/Cas systems. *Science.* 2013;339(6121):819–23. 10.1126/science.1231143 23287718 PMC3795411

[53] Voit RA Hendel A Pruett-Miller SM: Nuclease-mediated gene editing by homologous recombination of the human globin locus. *Nucleic Acids Res.* 2014;42(2):1365–78. 10.1093/nar/gkt947 24157834 PMC3902937

[54] Huang X Wang Y Yan W: Production of Gene-Corrected Adult Beta Globin Protein in Human Erythrocytes Differentiated from Patient iPSCs After Genome Editing of the Sickle Point Mutation. *Stem Cells.* 2015;33(5):1470–9. 10.1002/stem.1969 25702619 PMC4628786

[55] Xie F Ye L Chang JC: Seamless gene correction of β-thalassemia mutations in patient-specific iPSCs using CRISPR/Cas9 and *piggyBac*. *Genome Res.* 2014;24(9):1526–33. 10.1101/gr.173427.114 25096406 PMC4158758

[56] Sun N Zhao H: Seamless correction of the sickle cell disease mutation of the *HBB* gene in human induced pluripotent stem cells using TALENs. *Biotechnol Bioeng.* 2014;111(5):1048–53. 10.1002/bit.25018 23928856

[57] Ma N Liao B Zhang H: Transcription activator-like effector nuclease (TALEN)-mediated gene correction in integration-free β-thalassemia induced pluripotent stem cells. *J Biol Chem.* 2013;288(48):34671–9. 10.1074/jbc.M113.496174 24155235 PMC3843079

[58] Ramalingam S Annaluru N Kandavelou K: TALEN-mediated generation and genetic correction of disease-specific human induced pluripotent stem cells. *Curr Gene Ther.* 2014;14(6):461–72. 10.2174/1566523214666140918101725 25245091

[59] Wienert B Funnell AP Norton LJ: Editing the genome to introduce a beneficial naturally occurring mutation associated with increased fetal globin. *Nat Commun.* 2015;6:7085. 10.1038/ncomms8085 25971621

[60] Hoban MD Cost GJ Mendel MC: Correction of the sickle cell disease mutation in human hematopoietic stem/progenitor cells. *Blood.* 2015;125(17):2597–604. 10.1182/blood-2014-12-615948 25733580 PMC4408287

[61] Liang P Xu Y Zhang X: CRISPR/Cas9-mediated gene editing in human tripronuclear zygotes. *Protein Cell.* 2015;6(5):363–72. 10.1007/s13238-015-0153-5 25894090 PMC4417674

[62] Sun N Liang J Abil Z: Optimized TAL effector nucleases (TALENs) for use in treatment of sickle cell disease. *Mol Biosyst.* 2012;8(4):1255–63. 10.1039/c2mb05461b 22301904

[63] Lin Y Cradick TJ Brown MT: CRISPR/Cas9 systems have off-target activity with insertions or deletions between target DNA and guide RNA sequences. *Nucleic Acids Res.* 2014;42(11):7473–85. 10.1093/nar/gku402 24838573 PMC4066799

[64] Fu Y Foden JA Khayter C: High-frequency off-target mutagenesis induced by CRISPR-Cas nucleases in human cells. *Nat Biotechnol.* 2013;31(9):822–6. 10.1038/nbt.2623 23792628 PMC3773023

[65] Cradick TJ Fine EJ Antico CJ: CRISPR/Cas9 systems targeting β-globin and *CCR5* genes have substantial off-target activity. *Nucleic Acids Res.* 2013;41(20):9584–92. 10.1093/nar/gkt714 23939622 PMC3814385

[66] Ran FA Hsu PD Lin CY: Double nicking by RNA-guided CRISPR Cas9 for enhanced genome editing specificity. *Cell.* 2013;154(6):1380–9. 10.1016/j.cell.2013.08.021 23992846 PMC3856256

[67] Tsai SQ Wyvekens N Khayter C: Dimeric CRISPR RNA-guided FokI nucleases for highly specific genome editing. *Nat Biotechnol.* 2014;32(6):569–76. 10.1038/nbt.2908 24770325 PMC4090141

[68] Fu Y Sander JD Reyon D: Improving CRISPR-Cas nuclease specificity using truncated guide RNAs. *Nat Biotechnol.* 2014;32(3):279–84. 10.1038/nbt.2808 24463574 PMC3988262

[69] Smith C Gore A Yan W: Whole-genome sequencing analysis reveals high specificity of CRISPR/Cas9 and TALEN-based genome editing in human iPSCs. *Cell Stem Cell.* 2014;15(1):12–3. 10.1016/j.stem.2014.06.011 24996165 PMC4338993

[70] Veres A Gosis BS Ding Q: Low incidence of off-target mutations in individual CRISPR-Cas9 and TALEN targeted human stem cell clones detected by whole-genome sequencing. *Cell Stem Cell.* 2014;15(1):27–30. 10.1016/j.stem.2014.04.020 24996167 PMC4082799

[71] Wang X Wang Y Wu X: Unbiased detection of off-target cleavage by CRISPR-Cas9 and TALENs using integrase-defective lentiviral vectors. *Nat Biotechnol.* 2015;33(2):175–8. 10.1038/nbt.3127 25599175

[72] O'Geen H Henry IM Bhakta MS: A genome-wide analysis of Cas9 binding specificity using ChIP-seq and targeted sequence capture. *Nucleic Acids Res.* 2015;43(6):3389–404. 10.1093/nar/gkv137 25712100 PMC4381059

[73] Corrigan-Curay J O'Reilly M Kohn DB: Genome editing technologies: defining a path to clinic. *Mol Ther.* 2015;23(5):796–806. 10.1038/mt.2015.54 25943494 PMC4427885

[74] Li L Krymskaya L Wang J: Genomic editing of the HIV-1 coreceptor CCR5 in adult hematopoietic stem and progenitor cells using zinc finger nucleases. *Mol Ther.* 2013;21(6):1259–69. 10.1038/mt.2013.65 23587921 PMC3677314

[75] Mussolino C Alzubi J Fine EJ: TALENs facilitate targeted genome editing in human cells with high specificity and low cytotoxicity. *Nucleic Acids Res.* 2014;42(10):6762–73. 10.1093/nar/gku305 24792154 PMC4041469

[76] Li HL Fujimoto N Sasakawa N: Precise correction of the dystrophin gene in duchenne muscular dystrophy patient induced pluripotent stem cells by TALEN and CRISPR-Cas9. *Stem Cell Reports.* 2015;4(1):143–54. 10.1016/j.stemcr.2014.10.013 25434822 PMC4297888

[77] Tan EP Li Y Velasco-Herrera Mdel C: Off-target assessment of CRISPR-Cas9 guiding RNAs in human iPS and mouse ES cells. *Genesis.* 2015;53(2):225–36. 10.1002/dvg.22835 25378133

[78] Tebas P Stein D Tang WW: Gene editing of *CCR5* in autologous CD4 T cells of persons infected with HIV. *N Engl J Med.* 2014;370(10):901–10. 10.1056/NEJMoa1300662 24597865 PMC4084652

[79] von Levetzow G Spanholtz J Beckmann J: Nucleofection, an efficient nonviral method to transfer genes into human hematopoietic stem and progenitor cells. *Stem Cells Dev.* 2006;15(2):278–85. 10.1089/scd.2006.15.278 16646674

[80] Wiehe JM Ponsaerts P Rojewski MT: mRNA-mediated gene delivery into human progenitor cells promotes highly efficient protein expression. *J Cell Mol Med.* 2007;11(3):521–30. 10.1111/j.1582-4934.2007.00038.x 17635643 PMC3922358

[81] Leurs C Jansen M Pollok KE: Comparison of three retroviral vector systems for transduction of nonobese diabetic/severe combined immunodeficiency mice repopulating human CD34 ^+^ cord blood cells. *Hum Gene Ther.* 2003;14(6):509–19. 10.1089/104303403764539305 12718762

[82] Genovese P Schiroli G Escobar G: Targeted genome editing in human repopulating haematopoietic stem cells. *Nature.* 2014;510(7504):235–40. 10.1038/nature13420 24870228 PMC4082311

[83] Yu C Liu Y Ma T: Small molecules enhance CRISPR genome editing in pluripotent stem cells. *Cell Stem Cell.* 2015;16(2):142–7. 10.1016/j.stem.2015.01.003 25658371 PMC4461869

[84] Certo MT Ryu BY Annis JE: Tracking genome engineering outcome at individual DNA breakpoints. *Nat Methods.* 2011;8(8):671–6. 10.1038/nmeth.1648 21743461 PMC3415300

[85] Shahar OD Kalousi A Eini L: A high-throughput chemical screen with FDA approved drugs reveals that the antihypertensive drug Spironolactone impairs cancer cell survival by inhibiting homology directed repair. *Nucleic Acids Res.* 2014;42(9):5689–701. 10.1093/nar/gku217 24682826 PMC4027216

[86] Hendel A Kildebeck EJ Fine EJ: Quantifying genome-editing outcomes at endogenous loci with SMRT sequencing. *Cell Rep.* 2014;7(1):293–305. 10.1016/j.celrep.2014.02.040 24685129 PMC4015468

[87] Kuhar R Gwiazda KS Humbert O: Novel fluorescent genome editing reporters for monitoring DNA repair pathway utilization at endonuclease-induced breaks. *Nucleic Acids Res.* 2014;42(1):e4. 10.1093/nar/gkt872 24121685 PMC3874187

[88] Chu VT Weber T Wefers B: Increasing the efficiency of homology-directed repair for CRISPR-Cas9-induced precise gene editing in mammalian cells. *Nat Biotechnol.* 2015;33(5):543–8. 10.1038/nbt.3198 25803306

[89] Maruyama T Dougan SK Truttmann MC: Increasing the efficiency of precise genome editing with CRISPR-Cas9 by inhibition of nonhomologous end joining. *Nat Biotechnol.* 2015;33(5):538–42. 10.1038/nbt.3190 25798939 PMC4618510

[90] Lin S Staahl BT Alla RK: Enhanced homology-directed human genome engineering by controlled timing of CRISPR/Cas9 delivery. *eLife.* 2014;3:e04766. 10.7554/eLife.04766 25497837 PMC4383097

[91] Weatherall DJ Clegg JB: The Thalassaemia Syndromes. Blackwell Science Ltd.,2001;4 10.1002/9780470696705

[92] Byrne SM Ortiz L Mali P: Multi-kilobase homozygous targeted gene replacement in human induced pluripotent stem cells. *Nucleic Acids Res.* 2015;43(3):e21. 10.1093/nar/gku1246 25414332 PMC4330342

[93] Thein SL: Genetic modifiers of beta-thalassemia. *Haematologica.* 2005;90(5):649–60. 15921380

[94] Galanello R Cao A: Relationship between genotype and phenotype. Thalassemia intermedia. *Ann N Y Acad Sci.* 1998;850:325–33. 10.1111/j.1749-6632.1998.tb10489.x 9668554

[95] Sankaran VG Menne TF Xu J: Human fetal hemoglobin expression is regulated by the developmental stage-specific repressor *BCL11A*. *Science.* 2008;322(5909):1839–42. 10.1126/science.1165409 19056937

[96] Sankaran VG Xu J Ragoczy T: Developmental and species-divergent globin switching are driven by *BCL11A*. *Nature.* 2009;460(7259):1093–7. 10.1038/nature08243 19657335 PMC3749913

[97] Bauer DE Kamran SC Lessard S: An erythroid enhancer of *BCL11A* subject to genetic variation determines fetal hemoglobin level. *Science.* 2013;342(6155):253–7. 10.1126/science.1242088 24115442 PMC4018826

[98] Canver MC Smith EC Sher F: *BCL11A* enhancer dissection by Cas9-mediated *in situ* saturating mutagenesis. *Nature.* 2015;527(7577):192–7. 10.1038/nature15521 26375006 PMC4644101

[99] Corlan AD: Medline trend: automated yearly statistics of PubMed results for any query.2004 Reference Source

